# Plant Extracts and Bee Products as Bioactive Compounds: From Mechanism to Delivery

**DOI:** 10.3390/pharmaceutics18070882

**Published:** 2026-07-20

**Authors:** Radosław Balwierz, Ewelina Szliszka, Małgorzata Kłósek, Anna Kurek-Górecka

**Affiliations:** 1 Institute of Chemistry, University of Opole, Oleska 48, 45-052 Opole, Poland; 2 Department of Microbiology and Immunology, Faculty of Medical Sciences, Medical University of Silesia in Katowice, Jordana 19, 41-808 Zabrze, Poland; eszliszka@sum.edu.pl (E.S.); mklosek@sum.edu.pl (M.K.); akurekgorecka@sum.edu.pl (A.K.-G.)

## 1. Introduction

Plant extracts and bee products have accompanied pharmacotherapy for centuries and remain among the most productive sources of pharmacologically active substances, particularly in oncology and infectious diseases [1]. Their secondary metabolites, including alkaloids, flavonoids, carotenoids and terpenes, together with the peptides of bee venom, combine anti-inflammatory, immunomodulatory, antimicrobial, cardioprotective and anticancer activity, as repeatedly confirmed in in vitro and in vivo studies [1,2]. However, the path from a promising extract to a medicine meets the same recurring obstacles: poor solubility, low bioavailability, chemical instability and an incomplete understanding of the mechanism of action [1]. It is these obstacles, rather than any lack of biological activity, that most often halts natural substances at the preclinical stage.

The available responses follow two axes. The first is a deeper recognition of the mechanism of action, which allows the actual pharmacological effect to be separated from non-specific phenomena. The second is the rational design of the dosage form, in which nanostructured carriers improve the solubility, stability and bioavailability of the active substance and, at the same time, enable its controlled and targeted release [3]. At the intersection of these two axes, a natural product ceases to be an extract with promising properties and becomes a well-defined drug candidate.

This Special Issue, “Plant Extracts and Their Biomedical Applications”, gathered seven papers: four original articles and three reviews. The collected reports cover both of these axes. On the one hand, they show natural substances in action, across indications ranging from oncology, through cardioprotection and parasitic diseases, to orofacial pain. On the other, they illustrate the formulation and nanotechnological strategies on which the effective delivery of these compounds to the site of action depends.

## 2. Overview of Published Work

### 2.1. Natural Active Substances: From Oncology to Pain

The first research report concerns colchicine, an alkaloid with strong antimitotic activity, in a three-dimensional model of MCF-7 breast cancer [Contribution (1)]. The authors traced its influence on glycosaminoglycans (GAGs) and cell viability in a hollow-fiber bioreactor, using magnetic resonance imaging (MRI) with fixed charge density (FCD) and T1 relaxation mapping as a non-invasive quantitative tool. Colchicine reduced cell viability and altered the GAG content in both lines, HER-2 positive and HER-2 negative. The value of the work lies in coupling the action of a natural compound with the monitoring of extracellular matrix remodeling in a model of high physiological relevance.

Carotenoids from pumpkin pulp, extracted with natural deep eutectic solvents (NADES), became the subject of the second article, this time in the context of doxorubicin cardiotoxicity [Contribution (2)]. In a Wistar rat model, co-administration of the extract restored the Nrf2 expression suppressed by doxorubicin and normalized the apoptotic markers, caspase-3 and Bcl-2, as well as troponin I expression. The cardioprotective effect was linked to the Nrf2 pathway. It should be noted that a defined point of action was identified here, and not merely an observational correlation.

The third research paper shifts attention to parasitic diseases and evaluates the combination of curcumin with praziquantel in *Clonorchis sinensis* infection in rats [Contribution (3)]. A stronger antiparasitic effect than either compound alone was obtained, time- and dose-dependent in vitro, and in the rat model the combination reduced the worm burden together with liver fibrosis and bile duct dilation. Curcumin complements the reference drug here, and the benefit concerns not only the elimination of the parasite but also the tissue consequences of chronic infection. The work illustrates the logic of combination therapy based on a natural compound.

The clinical register changes with the fourth report, a review devoted to cannabidiol (CBD) in temporomandibular disorders (TMDs) and orofacial pain [Contribution (4)]. The authors discuss the anti-inflammatory and analgesic action mediated by CB1 and CB2 receptors and the reduction in cytokine release, noting at the same time that the clinical evidence remains preliminary and that well-designed randomized trials are lacking. The promising mechanism is, in this work, clearly separated from the shortage of conclusive clinical data.

### 2.2. Antiviral Phytochemicals and the Bioavailability Barrier

A separate thread of the issue is set by the review of the antiviral phytochemicals of etrog citron (*Citrus medica*), primarily flavonoids, coumarins, and terpenes [Contribution (5)]. The authors discuss the mechanisms of antiviral action with translational limitations, such as poor solubility and low bioavailability, and point directly to delivery strategies as the condition for turning these compounds into drug candidates. In this work, the two threads of the issue, biological activity and the manner of its delivery, are considered jointly rather than separately.

### 2.3. Nanotechnology as a Strategy to Improve Natural Products Delivery

The formulation plane of the issue is opened by a nanofilm combining bee venom (apitoxin) with poly(vinyl alcohol) and zinc oxide nanoparticles [Contribution (6)]. Mass spectrometry revealed 84 active components of the bee venom, and the formulation itself increased antidiabetic activity, measured by inhibition of alpha-amylase and alpha-glucosidase, as well as anti-inflammatory, antimicrobial and antioxidant activity. Of note, the nanofilm proved less toxic to HepG2 liver cells than the venom alone, with IC50 values of 52.27 and 18.5 µg/mL, respectively. The carrier therefore modified not only the availability but also the safety profile of the natural substance [2].

The issue closes with an extensive review of metallic nanoparticles synthesized with the participation of plant secondary metabolites, that is, by green synthesis [Contribution (7)]. The authors set out the advantages of the biogenic route, lower cost, reduced energy consumption and biocompatibility, against applications in cancer, diabetes, Alzheimer’s disease and vector-borne diseases. As one of the most frequently cited papers of this issue, it reflects the growing interest in sustainable, plant-based nanotechnology.

## 3. Future Perspectives

The collected papers outline three directions for further research in this area.

The first is the standardization and quality control of extracts. Reproducibility of biological effects requires a defined chemical profile of the raw material and validated extraction methods, including environmentally friendly solvents such as NADES. Without this, the comparability of results between laboratories remains limited.

The second is the transition from preclinical data to conclusive clinical trials. For cannabidiol or curcumin, the mechanism of action is increasingly well understood, whereas well-designed randomized trials that would establish efficacy, dosing and long-term safety are lacking. This gap is the one that subsequent work should address first.

The third and, from the perspective of this issue, the most important, is the rational design of the dosage form. Nanostructured carriers, including biogenically synthesized systems, improve solubility and bioavailability and at times modify the safety profile of the active substance [3]. For a result obtained in vitro to translate into a clinical effect, however, the in vitro release must be linked to in vivo availability—that is, in vitro–in vivo correlation (IVIVC)—together with an assessment of pharmacokinetics in translational models and a sound evaluation of nanomaterial safety. The therapeutic potential of natural substances depends on the combination of biological activity, a well-understood mechanism of action, and an appropriate formulation.

## Data Availability

No new data were created or analyzed in this study. Data sharing is not applicable to this article.
